# Comparative evaluation of selected concentrations of sodium hypochlorite on the outcome of endodontic therapy among Ghanaians

**DOI:** 10.1371/journal.pone.0306693

**Published:** 2024-07-08

**Authors:** Akua B. Konadu, Patrick C. Ampofo, Moses L. Akyeh, Sandra A. Hewlett, Kofi Osei-Tutu, Ebenezer A. Nyako

**Affiliations:** 1 Department of Restorative Dentistry, University of Ghana Dental School, College of Health Sciences, University of Ghana, Accra, Ghana; 2 Department of Bacteriology, Noguchi Memorial Institute for Medical Research, University of Ghana, Accra, Ghana; 3 Dental Department, the Trust Specialist Hospital, Accra, Ghana; University of Puthisastra, CAMBODIA

## Abstract

**Background:**

Endodontic treatment is one of the main dental treatments to manage inflamed or infected root canal systems of teeth. The success of endodontic treatment principally depends on eradicating microorganisms in the root canal by chemo-mechanical debridement with irrigation solutions like sodium hypochlorite (NaOCl). NaOCl has been used in concentrations ranging from 0.5% to 5.25%. This study determined the antimicrobial effectiveness of selected concentrations (0.5%, 1.0%, 2.6%, and 5.2%) of NaOCl in endodontic treatment.

**Methods:**

The study sites were the University of Ghana Dental School (UGDS) and Noguchi Memorial Institute for Medical Research (NMIMR). Sixty infected single-rooted single-canal teeth were used. Before (S_1_) and after (S_2_), root canal samples during the endodontic treatment with the selected concentrations of NaOCl were examined via anaerobic and aerobic cultures. The isolates were identified using Matrix Assisted Laser Desorption Ionization-Time Of Flight Mass Spectrometry (MALDI-TOF MS).

**Results:**

All S_1_ samples were positive for cultivable bacteria. Fifty-three (53) different microbial species belonging to 20 different microbial genera were isolated. *Streptococcus viridans* was the most frequently isolated microbe. There were zero isolates in the root canals irrigated with 2.6% and 5.2% NaOCl. Two teeth had isolates in the groups irrigated with the lower concentrations (0.5% and 1.0%) of NaOCl. The persistent bacteria were one species each of *Streptococcus mitis* and *Streptococcus oralis*, respectively.

**Conclusion:**

Root canal treatments using chemo-mechanical preparation with the selected concentrations (0.5%, 1.0%, 2.6%, and 5.2%) of NaOCl were effective in significantly reducing the microbial load, and for the 5.2% and 2.6% concentrations, in eliminating all the microorganisms.

## Introduction

Endodontics, or root canal treatment, is one of the main dental treatments offered to dental patients globally and in Ghana to manage infected and inflamed teeth. Endodontic treatment is a sequence of treatments used to control pulpal infection [1]. Endodontic treatment success primarily depends on eradicating microorganisms using chemo-mechanical debridement with irrigation solutions such as NaOCl and inter-appointment dressing with root canal medications [2, 3].

The use of irrigating solutions with mechanical instrumentation during endodontic procedures is an important process and can adequately disinfect root canal systems during endodontic therapy [4]. An irrigant with significant antibacterial action and minimal toxicity is recommended [5]. The most popular irrigant used in endodontic treatments is NaOCl solution. [5] It is affordable and has the ability to dissolve organic materials, as well as its antimicrobial properties. However, it can also irritate nearby periradicular tissues, causing pain and causing when accidentally extruded beyond the apex into surrounding tissues [5–7]. There is ongoing discussion over the ideal concentration of NaOCl for use in endodontics, and a range of 0.5% to 6.0% has been suggested [8].

In terms of root canal disinfection, NaOCl is effective against root canal bacteria such as Actinomyces naeslundi (found in untreated necrotic root canals), Enterococcus faecalis, and Candida albicans (found in endodontic failure cases) [9]. However, some clinical trials have found that even at a high concentration of 5%, roughly one-third to half of the root canals remain contaminated following instrumentation and irrigation with NaOCl [10]. A study conducted in Ghana discovered that root canal infections are polymicrobial, with facultative anaerobes predominating. Streptococcus spp., Prevotella spp., Actinomyces spp., Enterococcus faecalis, and Rothia spp. were among the bacteria often isolated [11]. Some studies discovered no significant difference in the antibacterial action of 1%, 2.5%, and 5.25% NaOCl in infected canals of removed teeth [8, 12].

A study showed 73.7% of Ghanaian dentists used NaOCl, routinely in various concentrations0.5% to 5.0% and 6.7%) during endodontic treatment in most dental clinics in Ghana [13]. Some of the practitioners in Ghana had experienced complications when using NaOCl, irrespective of the chosen concentration [13]. No study has been carried out in Ghana on the effective concentration of NaOCl that has good therapeutic and antimicrobial effects with minimal or no complications in root canal treatment. Most of the available data and recommendations are obtained mainly from research outside the country and sub-region. Though various concentrations of NaOCl have been used in studies across literature sources, none has been documented in Ghana, where different socio-economic situations and access to dental care influence treatment outcomes. Various factors, including geographic location, socio-economic status, food habits, and dental hygiene status [14], have influenced the kind and frequency of microbiological agents implicated in the pathophysiology of root canal infections. In Ghana and sub-Saharan Africa, where patients often seek dental care late due to socio-economic reasons and the unavailability of dental services and specialized care in some communities. it is essential to research and document the effectiveness of different concentrations of sodium hypochlorite since it is the most commonly used irrigation solution. This study selected (0.5%, 1.0%, 2.6%, and 5.2%) concentrations of NaOCl based on the commonly used concentration in Ghana and available literature to compare the antimicrobial effectiveness on root canal microorganisms eradication during endodontic treatment. This will help set protocols for using NaOCl in endodontic treatment and provide data to help decide the best approaches to improve outcomes.

## Materials and methods

This prospective study compared the effectiveness of selected concentrations of NaOCL during endodontic treatment. Data was collected from 30 ^Sept^ 2016 to 30 ^Jul^ 2017 at the endodontic clinic in the Restorative Dentistry Department of the University of Ghana Dental School. The study samples were teeth diagnosed with root canal infections. The Ethical and Protocol Review Committee of the College of Health Sciences of the University of Ghana provided its ethical approval. (Reference Number: CHS-Et/M.1-P 4.9/ 2016–2017).

The purpose of the study and potential risks and benefits were provided to willing participants through the study information leaflet. Interested participants were allowed to ask questions for clarification before completing the informed consent forms. Informed consent was obtained from all participants before the start of the study. Participants were informed of their right to withdraw from the study and have their data removed. Participants’ identities were anonymized to ensure confidentiality. Only participants who met the inclusion criteria and consented to the study were included.

### Sample size determination

The sample size calculation was calculated using Eng J’s equation. [15] The primary effect measure based on the study’s primary objective was a reduction in microbial load (colony-forming units). The pre-study percentage estimate was set at 53% and 99% for 0.5% and 5.2% concentrations of sodium hypochlorite, respectively, based on the study by Squiera et al. [12],. Using a 46% reduction between the higher and lower concentrations of the irrigation solutions at a 95% confidence level, 80% power, and a margin of error of 5%, the sample size was calculated to be 60 teeth with 5% attrition rate

$$N=2\;x\frac{{\left[Z_{crit}\sqrt{2\;\bar{p}(1-\bar{p})}+Z_{pwr}\sqrt{P_{1}(1-P_{1})+P_{2}(1-P_{2})}\right]}^{2}}{D^{2}}$$

The formula was modified to calculate the sample size for the four groups, as shown below.


$$N=4\;x\frac{{\left[Z_{crit}\sqrt{2\;\bar{p}(1-\bar{p})}+Z_{pwr}\sqrt{P_{1}(1-P_{1})+P_{2}(1-P_{2})}\right]}^{2}}{D^{2}}$$

Inputting the values in the equation, the sample size was calculated as follows.


$$N=4\;x\frac{{\left[1.96\sqrt{20.76(1-0.76)}+0.842\sqrt{0.99(1-0.99)+0.53(1-0.53)}\right]}^{2}}{0.46^{2}}$$

### Case selection

Single-rooted, single-canal teeth at different stages of endodontic diagnosis were selected from the endodontic clinic of the University of Ghana Dental School. The target population was patients 18 years and above with fully formed permanent teeth requiring non-surgical endodontic treatment referred to the endodontic clinic of the University Ghana Dental School. Patients who had taken antibiotics within a month before the presentation and teeth with root anomalies, periodontal conditions, and poor restorative prognosis were excluded. Teeth with secondary infection requiring re-treatment were also excluded from the study.

A semi-structured questionnaire was used to obtain the participants’ general condition, demographic data, and tooth characteristics. The soft tissue status, tooth mobility, and coronal and radicular restorations in the patient’s oral cavity were assessed and recorded on clinical examination forms. The presence or absence of periradicular radiolucency and response to both electric and cold pulp testing were also recorded.

Each tooth used for the root canal treatment was examined and assessed per the inclusion criteria. The pulpal diagnosis was based on clinical examination, pulp sensibility testing, and radiographs, which were used to check for periapical lesions. [16]

The teeth selected for this study were diagnosed based on their clinical presentation and symptoms, response to both electric and cold pulp testing, and radiographic findings. The tenth edition of the American Association of Endodontists glossary of endodontic conditions was used to categorise the diagnosis. *Normal Pulp* is a clinical diagnostic category in which the Pulp is symptom-free and responds normally to pulp testing. In *Irreversible Pulpitis*, there was pulpal exposure from either caries or trauma, and the tooth responded to pulp testing; in *pulpal necrosis*, the tooth may be discoloured, the Pulp is non-responsive to pulp testing, and it is asymptomatic with no periapical radiolucency. *Symptomatic Apical Periodontitis*: there are clinical symptoms, positive response to percussion or palpation, may or may not be accompanied by radiographic changes (i.e., depending on the stage of the disease, the periodontal ligament may be normal width or there may be periapical radiolucency). *Asymptomatic Apical periodontitis*: the Pulp is non-responsive, there is no pain to percussion test, there are radiographic changes, and the tooth is asymptomatic. An a*cute apical abscess* was an episode of rapid onset of swelling and pain and non-responsiveness to pulp testing, with or without periapical radiolucency. *Chronic apical abscess;* tooth with chronic swelling non-responsive to pulp testing, with periapical radiolucency [17].

### Randomization

A total of 60 teeth were used in the study, and these were from 44 individuals. Using the balloting method, the investigators randomly assigned the teeth into four(4)groups of 15 teeth each corresponding to a different concentration of NaOCl. For participants with more than one tooth suitable for the study, the first tooth was placed in the group picked by balloting, and the subsequent teeth were allocated to the next consecutive letter picked to ensure systematic assignment.

### Root canal treatment and microbial sample collection

The root canal treatment and sample collection were done by the same clinician (principal investigator) under aseptic conditions using a rubber dam to ensure standardisation of the root canal treatment and eliminate operator bias and variability. According to Peters et al. protocols, the access cavity was cleansed with methylated spirit before entering the pulp chamber [18]. Root canal samples were collected before (S1) and after (S2) treatment for microbiological analysis. The samples collected for microbiological analysis were placed in a tube containing Phosphate Buffered Solution (PBS) using a sterile paper point (from Henry Schein).

The radiographic method was used to determine the working length. The root canals were prepared using Pro Taper (Dentsply Sirona) hand files, the crown down technique, and extensive irrigation with the appropriate concentrations of NaOCl solution and normal saline. Irrigants were administered into the root canal using a 23 gauge blind-ended needle with a side vent via traditional irrigation [19]. The maxillary teeth were cleaned to F3, and the mandibular incisors to F2 finishing files. Gutta-percha matching the finishing files F3 and F2 was used with a root canal sealant (Sealapex) to obturate the root canals.

Sodium Hypochlorite was used to irrigate the root canal copiously during the treatment. After the root canal had been cleaned and shaped, it was filled with a selected concentration of NaOCl and left alone for 5 minutes. Immediately after this, the canal was continually irrigated with an additional 5 ml of the chosen concentration of NaOCl. The canal was finally irrigated with 10 ml of normal saline. The second microbial sample (S2) was collected from the root canal following the procedures above using the sterile paper point (ISO sizes 15–25) mentioned above for microbial analysis.

The canal was considered entirely clean When the file felt snug along its entire working length, and no debris was visible at the tip upon inspection. A cotton pellet was put in the pulp chamber after the canal had been dried and dressed with non-setting calcium hydroxide. Glass Ionomer cement was then used to restore the access cavity.

Participants were asked to use a visual analog scale (VAS) to record their preoperative pain before receiving a local anaesthetic. For seven days after the intervention, participants were instructed to record their pain on a 10-cm VAS scale, with 0 representing no pain and 10 representing the highest level of agony imaginable. Patients brought the completed surveys back to the clinic one week later. For better clinical interpretability, pain on the VAS was further divided into three categories: mild (1–3), moderate (4–6), and severe (7–10). The teeth were obturated during the second visit with Gutta-percha and Sealapex (Dentsply), and the access was restored.

### Isolating and detection of species

Laboratory procedures followed the CLSI (Clinical and Laboratory Standard Institute) guidelines. To avoid delays in processing the samples, a laboratory setup was constructed on the chair side of the dental clinic to begin processing. The processed specimen was plated in an anaerobic jar, carried to the laboratory in a mobile incubator for 2 hours, and then placed in a laboratory incubator at 37oC for 18–24 hours in the air for further processing. The samples (paper points) were vortexed in 2mL of PBS for 45 seconds using an Eltek VM 301 vortex mixer. Buffered Peptone Water was used to prepare 10-fold serial dilutions of the bacterial suspensions. 0.1 ml dilution aliquots were used to inoculate non-selective enriched Anaerobe Basal Blood Agar (ABBA) primary isolation plates, and they were disseminated with a sterile bent plastic rod. The anaerobic culture medium consisted of anaerobe basal agar with an addition of 5% defibrinated sheep blood plates. It was maintained in an anaerobic jar (Becton Dickinson) with an anaerobic generation kit containing 85% N2, 10% H2, and 5% CO2 (Oxoid Unipath Ltd., Basingstoke, Hampshire, UK) at 37°C for 48–72 hours. Daily checks were made for growth on all plates. After 48 hours, the anaerobic jar was unsealed.

For aerobic culture, samples were streak-plate inoculated on Sheep Blood agar, selective media Mac Conkey agar (Oxoid Unipath Ltd., Basingstoke, Hampshire, UK), and Uri Select agar (Bio-Rad), and kept in a mobile incubator for 2 hours before being transported to the lab and finally placed in an incubator at 37°C for 18–24 hrs in air. All of the methods used to identify these microorganisms were carried out following the recommendations of the CLSI (Clinical and Laboratory Standard Institute). Each plate was examined after incubation, and the various colonies were subcultured and identified [14].

### Microbial identification

The Bruker Biotype Matrix-Assisted Laser Desorption/Ionization Time-Of-Flight Mass Spectrometer (MALDI-TOF MS) equipment (Bruker Daltonics GmbH, Leipzig, Germany) was used to identify all the bacteria isolated in this study. All colonies from anaerobic and aerobic culture plates were purified into pure cultures and identified by MALDI-TOF. The Bruker Biotype MALDI-TOF MS system includes the Microflex LT/SH MS instrument and two software programs: FlexControl for the acquisition of protein spectra and Biotyper real-time classification (RTC) for automated spectral analysis. MALDI-TOF mass spectrometry (MS) has various advantages; for example, once a mass spectrometer and databases are accessible in a laboratory, identifying particular pathogens is cost-effective. Sample preparation is not technique-sensitive and does not require extensive equipment. It provides greater diagnostic accuracy, robustness, dependability, and fast turnaround time and is very resistant to contamination [20]. The MALDI_TOF equipment was calibrated using bacterial test standard was used as a quality control recommended by the manufacturer. For internal accuracy control, Gram-negative rod-shaped Escherichia coli (ATCC 25922) and Gram-positive rod-shaped Listeria monocytogenes (ATCC 35152) were used as standard strains to augment the quality control of the Maldi-tof identification system. The same laboratory technician performed the microbial isolation and identification.

### Data analysis

Data were captured with Microsoft Access 2013 and analysed using the Statistical Package for Social Sciences (SPSS) version 22. The pain rating and the colony-forming units were skewed, so the Wilcoxon and Kruska-Wallis T-tests were used to compare the median among the various concentrations of NaOCl and clinical diagnoses. The chi-square test was used to compare the pain categories among the concentrations. Statistical significance was set at P<0.05.

### Ethics statement

The study was conducted following the protocols and guidelines of the Declaration of Helsinki. Ethical approval was obtained from the Ethical and Protocol Review Committee of the College of Health Sciences of the University of Ghana. (Reference Number: CHS-Et/M.1-P 4.9/ 2016–2017).

#### Informed consent statement

Written informed consent was sought from study participants before data collection. They were told of their freedom to decline participation without any consequences. Participants were also informed that they were free to withdraw from participating in the study at any time. Privacy and confidentiality:

Regarding privacy and anonymity, the information gathered has been treated with strict confidentiality. All information and data records were coded with an ID number and no identifying information was kept in the medical records. All participants were given the opportunity to ask any questions they had about the study before signing the informed consent.

### Results

Sixty teeth were used for the root canal therapy, which came from 44 patients. The age of the patients ranged from 20 to 75 (mean age 40.3 ± 14.9 years). Of the 44 patients who participated in the study, 32 (72.7%) were in the 20–40 age group.

**Fig 1:**
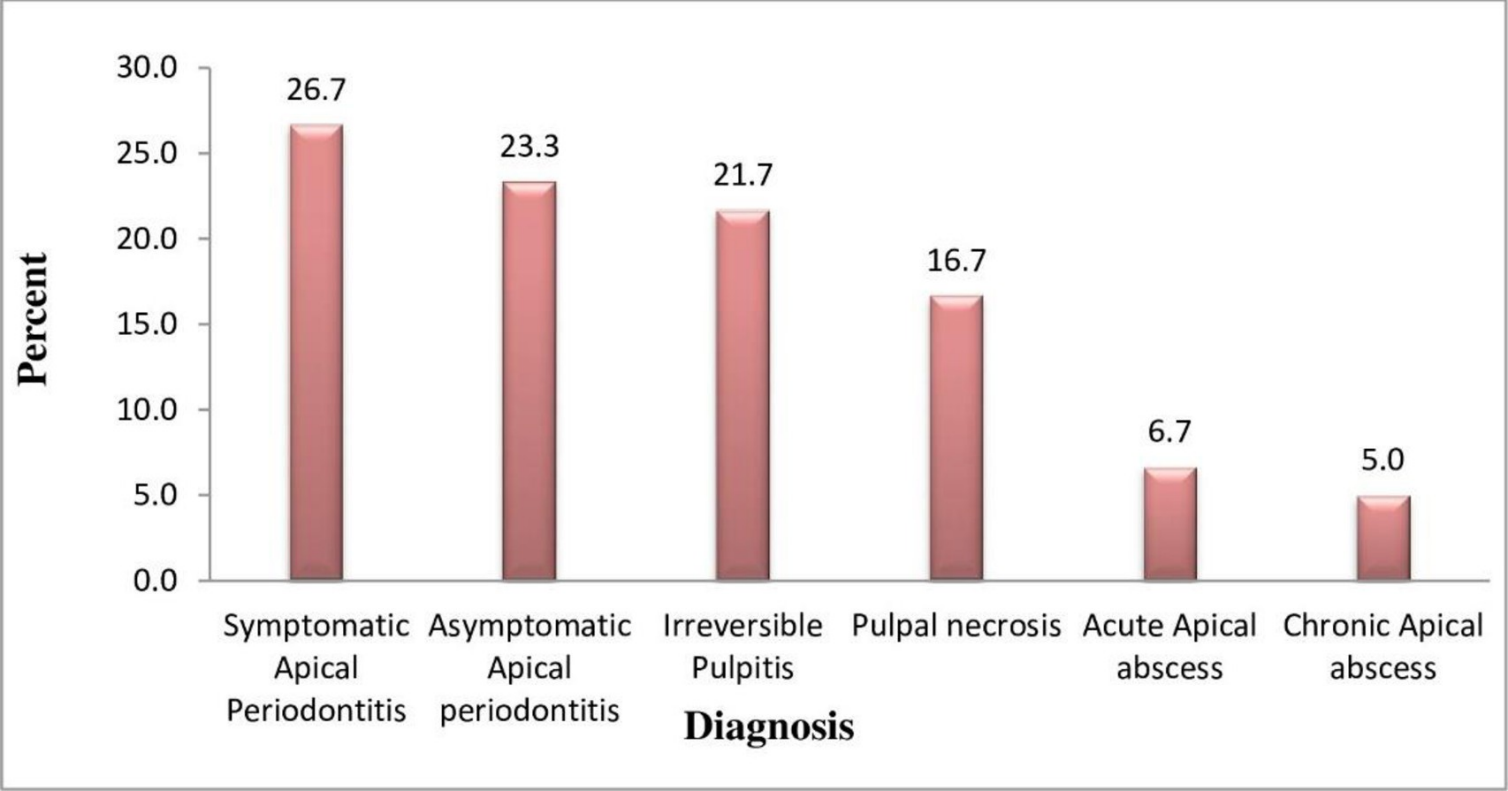
Diagnosis of teeth used for the study.

A total of 60 teeth were used for the root canal treatment, of which 31 (51.7%) were upper central incisors, 9 (15%), upper lateral incisors 4 (6.7%), upper canines lower 4 (6.7%), central lateral incisors, 3 (6.7%), lower canines, 1 (1.7%) lower first and 4 (6.7%) second premolars. The diagnosis of the teeth used is shown in Figure (Fig 1). Out of all 60 teeth used for the study, symptomatic apical periodontitis was diagnosed in 16 (26.7%) teeth, and asymptomatic apical periodontitis in 14 (23.3%)teeth and 10 (16.7%) teeth were found to have palpable necrosis, and 13 (21.7%) were found to have irreversible pulpitis.

### Microbial analysis

#### Microbial load (colony forming unit. {Cfu/ml})

The pre-treatment bacterial colony-forming unit (CFU) values varied from 10^6^ to 10^4^, while the post-treatment values ranged from 0 to 10^3^. Because the distribution was skewed, a Wilcoxon and Kruska-Wallis T-test was used to compare the median among the various concentrations of NaOCl and clinical diagnoses, which is reported in Table 1.

**Table 1:** Relationship between colony-forming units (cfu/ml) and the different concentration groups of NaOCl used for irrigation.

Concentration of Irrigant (n = 15) and Statistics		Colony Forming Unit (cfu) /ml Before Treatment x10^4^	Colony Forming Unit (cfu) /ml After Treatment x10^2^	*Wilcoxon Signed P value*
0.50%	Mean± SD	46.3 ±82.8	7.46 ± 29.0	***0*.*001***
	Median	2.50	0	
1.00%	Mean ± SD	98.1 ±18.7	1.06 ± 4.13	***<0*.*001***
	Median	1.86	0	
2.60%	Mean ± SD	63.1 ±91.3	0	***0*.*001***
	Median	23.1	0	
5.20%	Mean ± SD	20.0 ±32.0	0	***0*.*001***
	Median	8.50	0	
Total	Mean ± SD	34.8 ±66.2	2.13 ± 14.6	***<0*.*001***
	Median	5.95	0	
***P Valve***	***Kruskal-Wallis***	**0.298**	**0.565**	

Colony forming units (cfu/ml) significantly decreased in the treated groups (Wilcoxon signed-rank test, p 0.05). However, according to the Kruskal-Wallis test, there was no significant difference between the distribution of the colony-forming units across the various concentrations before and after p = 0.298 and p = 0.565.

In the current study, root canal samples (S1), before treatment, were found to contain at least two bacterial species. The most bacterial species identified per canal were nine, which were related to an acute apical abscess diagnosis.

The highest cfu/ml was 3.0x106 cfu/ml, related to symptomatic apical periodontitis and a pain (VAS) score of 2 (mild pain). The diagnosis of asymptomatic apical periodontitis had the lowest cfu/ml of 3.0x103 /ml.

The pre and post-treatment microbial load (cfu/ml) had means and standard deviations of 3.48 x 105 ± 6.62 x 105 and 2.13 x 102 ± 1.46 x 102, respectively.

#### Effect of the selected concentrations of NaOCl on colony forming units (cfu/ml)

There was no statistically significant difference in the microbial colony-forming units (cfu/ml) between the various concentrations of NaOCl used before(p = 0.72) after (p = 1.00) treatment. This suggests that while NaOCl effectively reduced microbial load overall, the concentrations did not significantly influence this reduction.

**Fig 2:**
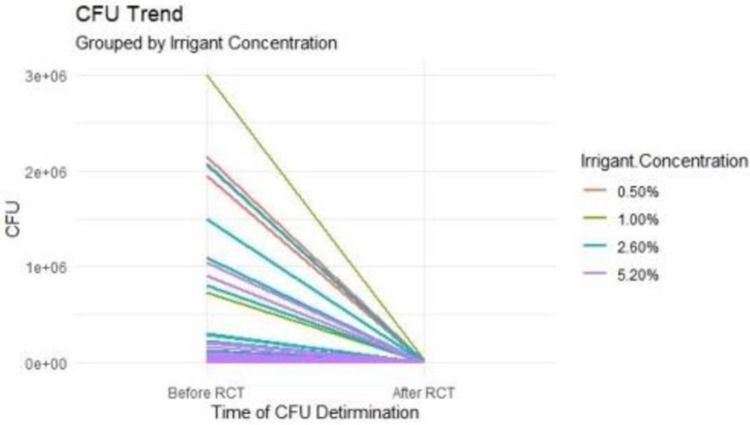
Effect of the different concentrations of NaOCl on colony forming units cfu/ml.

Fig 2 and Table 1 show the effects of the selected concentrations of NaOCl on colony-forming units.

#### Microbial load (type of microbes isolated from infected canals before treatment)

*Genera of microorganisms isolated from the root canals before treatment*. Twenty microbial genera (19 bacterial and a fungus) were recovered from the 60 infected root canals. Of the 20 different microbial genera isolated, seven organisms were obligately anaerobic, seven were facultatively anaerobic/aerobic, and five were aerobic. A fungus (Candida albicans) was also isolated. Gram-positive bacteria made up ten of the isolated bacteria genera, while gram-negative bacteria made up the rest.

The different microbial species, their Gram stain, morphology, oxygen tolerance, and the number of teeth they were isolated from are shown in **Table 2.**

**Table 2:** Microbial species isolated from infected root canals before treatment.

Microbial Species	Number Isolated	Gram Stain/Morphology	Bacteria Species	Number Isolated	Gram Stain/Morphology
**ANAEROBES**					
**Prevotella**	**(19)**	**- Rod**	**Fusobacterium**	**(6)**	**- Rod**
*P*.*buccae*	5	-Rod	*F nucleatum*	4	-Rod
*P*. *denticola*	4	- Rod	*F*.*periodonticum*	2	- Rod
*P*. *intermedia*	4	-Rod	*Veillonella parvula*	5	-cocci
*P*. *marshii*	2	- Rod	**Actinomyces**	**(11)**	**+Rod**
*P*. *oralis*	2	-Rod	*Actinomyces meyeri*	3	+Rod
*P*. *loescheii*	1	- Rod	*Actinomyces odontolyticus*	8	+Rod
*P*. *oris*	1	-Rod	*Lactobacillus mucosae*	1	+Rod
*Slackia exigua*	3	+Rod	*Propionibacterium acnes*	2	+Rod
*Parvimonas micra*	2	+cocci			
**FACULTATIVE ANAEROBES**					
**Streptococcus**	**(103)**	**+cocci**	*S*. *haemolyticus*	2	+cocci
*S*. *oralis*	22	+cocci	*S*. *cristatus*	1	+cocci
*S*.*mitis*	10	+cocci	*S*. *australis*	1	+cocci
*S*. *constellatus*	10	+cocci	*S*.*anginosus*	1	+cocci
*S*. *salivarius*	9	+cocci	*Enterococcus faecalis*	2	+cocci
*S*. *sanguinis*	7	+cocci			
*S*. *mutans*	6	+cocci	*Corynebacterium amycolatum*	1	+ bacillus
*S*. *parasanguinis*	6	+cocci	**Rothia species**	**(11)**	**+ coccobacillus**
*S*. *anginosus*	5	+cocci	*R*. *dentocariosa*	6	+ coccobacillus
*S*. *gordonii*	5	+cocci	*R*. *mucilaginosa*	4	+ coccobacillus
*S*. *infantis*	5	+cocci	*R*. *aeria*	1	+ coccobacillus
*S*. *sanguis*	4	+cocci	**Actinomyces**	**(5)**	**+ Rod**
*S*. *cristatus*	3	+cocci	*A*. *naeslundii*	4	+ Rod
*S*. *pneumoniae*	3	+cocci	*A*. *radicidentis*	1	+ Rod
*S*. *infantis*	3	+cocci			
**AEROBES**					
**Streptococcus**	**(43)**	**+cocci**	Micrococcaceae		+cocci
*S*.*mutans*	12	+cocci	*M*.*luteus*	1	+cocci
*S*. *oralis*	12	+cocci	Rhodococcus		+cocci
*S*. *mitis*	10	+cocci	*R*. *rhodochrous*	3	+cocci
*S*. *cristatus*	4	+cocci	**Neisseria**	**(6)**	**-diplococci**
*S*.*constellatus*	3	+cocci	*N*. *flavescens*	4	-diplococci
*S*. *sanguinis*	2	+cocci	*N*. *subflava*	2	-diplococci
*Enterococcus faecalis*	(14)	+cocci	*Escherichia coli*	2	-Rod
**Staphylococcus**	**(8)**	**+cocci**	*Pseudomonas stutzeri*	1	-Rod
*S*. *epidermidis*	4	+cocci	**Enterobacter**	**(8)**	**-Rod**
*S*. *warneri*	1	+cocci	*E*.*cloecae*	7	-Rod
*S*. *saprophyticus*	1	+cocci	*E*. *kobei*	1	-Rod
*S*. *haemolyticus*	2	+cocci			

There were 53 different bacterial isolates, and two isolates were Fungi (Candida albicans). Fifty-three microbial species were isolated and belonged to 20 different microbial genera.

The isolate of 53 bacteria was made up of 26 (49.1%) obligatory anaerobes, 15 (28.3%) facultative anaerobes, and 12 (22.6%) aerobes.

According to gram staining characteristics, 37 (69.8%) species were gram-positive species, and 16 (30.2%) were gram-negative. The most predominant genera isolated was streptococcus, followed by Prevotella and Rothia. The common streptococcus species that were isolated *were Streptococcus oralis*, *S*. *mitis*, *S*. *mutans*, *and S*. *constellatus*.

The study identified a diverse range of microbial species before treatment, with obligately anaerobic species being the most prevalent. After treatment, there was a significant reduction in microbial counts across all concentrations of NaOCl, indicating the effectiveness of root canal therapy in reducing microbial load.

#### Microbial species isolated after treatment

All concentrations of NaOCl showed a significant reduction in microbial counts post-treatment, with a maximum reduction in the 2.6% and 5.2% NaOCl groups that did not show any isolates post-treatment. One species of *Streptococcus mitis* and *S*.*oralis* was isolated from one tooth in each group irrigated with 1.0% and 0.5% NaOCl, with a diagnosis of acute apical abscess and symptomatic apical periodontitis, respectively, post-treatment.

#### Pain ratings using visual analog scale (VAS) scores

There was a significant reduction in pain intensity and frequency after root canal treatment with different concentrations of NaOCl. Most participants reported no pain after treatment, indicating the effectiveness of the procedure in alleviating pain. However, there was no significant difference in pain outcomes between the different concentrations of NaOCl used.

The intensity and frequency of pain experienced by participants were recorded using the pain VAS scores. Pain VAS scores of zero were categorized as no pain, 1–3 as mild pain, and 4–10 as Moderate–severe pain. The Pain (VAS) scores before varied from moderate to severe 21 (35.0%) and mild (12 (20%). Only 11(18.3%) teeth experienced mild pain after the treatment; the rest, 49 (81.7%)experienced No pain.(Table 3) The participants who experienced mild pain after treatment did so within 24 hours after the first treatment session, and the pain was eliminated after 48 hours. Comparing the proportion of people with pain outcomes between the various concentrations of NaOCl, it was not statistically significant using the chi-square test at a p 0.05 (p = 0.696).

**Table 3:** Pre and post-treatment pain (VAS) scores.

Vas grading	VAS grading before treatment		VAS grading after treatment	
	Number(n)	Percent (%)	Number (n)	Percent (%)
No pain	27	45.0	49	81.7
Mild	12	20	11	18.3
Moderate- severe	21	35.0	0	0
Total	60	100	60	100

The frequency and severity of pain were significantly reduced by the various NaOCl concentrations after the root canal therapy.

Comparison of Pre and Post-treatment Pain VAS Scores: The analysis showed a statistically significant difference (Wilcoxon signed rank test p<0.001) in pre-treatment and post-treatment pain VAS scores, indicating a reduction in pain levels following root canal therapy. This reduction was consistent across all concentrations of NaOCl. The pre and post-treatment pain VAS scores’ mean and standard deviations are 2.58 ± 3.066 and 0.30 ± 0.696, respectively, as shown in Table 4.

**Table 4:** Descriptive characteristics of the pain VAS scores among the different concentration groups of NaOCl before and after treatment.

Concentration of NaOCl (n = 15)	VAS Before Treatment	VAS After Treatment	Wilcoxon
	**Mean ± SD**	**Mean ± SD**	**P value**
0.5%	1.87 ± 3.25	0.27 ± 0.70	0.26
1.0%	3.2 ± 3.21	0.2 ± 0.56	0.005
2.6%	2.93 ± 2.94	0.2 ± 0.56	0.005
5.2%	2.33 ± 3.00	0.53 ± 0.92	0.15
Total	2.58 ± 3.07	0.3 ± 0.70	<0.001
Kruskal-Wallis	0.353	0.430	
P Valve			

**Fig 3:**
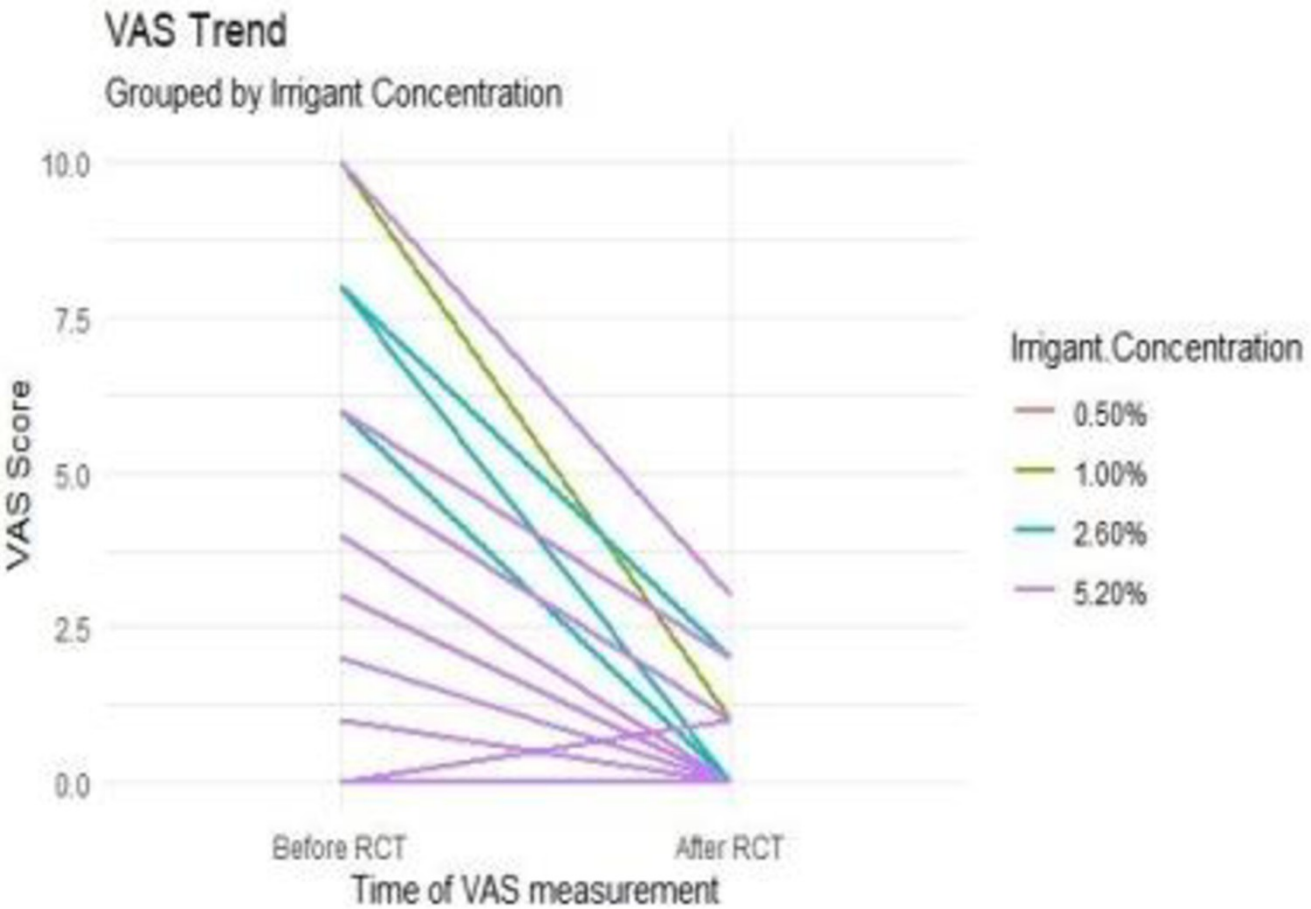
Relationship between pain VAS grades among the different concentration groups of NaOCl before and after treatment.

There was a general reduction in pain levels after treatment. (Fig 3) There was no significant difference in the incidence of pain between the different concentrations of NaOCl before and after treatment. P < 0.05 (Independent Kruskal-Wallis; before (p = 0.353) and after (p = 0.430).

## Discussion

This study compared the effects of different concentrations of NaOCl (0.5%, 1.0%, 2.6%, and 5.2%) on the result of endodontic therapy. The NaOCl solutions effectively removed microorganisms and reduced colony-forming units (cfu/ml) in the root canals.

This in-vivo study gives valuable evidence about the influence of NaOCl on colony-forming units and isolated root canal bacteria. When compared to alternative diagnostic tools such as polymerase chain reaction (PCR) assays, MALDI-TOF mass spectrometry (MS) has the benefit of being less expensive in low and middle-income countries such as Ghana, where resources are limited. It also provides greater diagnostic accuracy, robustness, dependability, and fast turnaround time, and is very resistant to contamination [20].

Due to its antibacterial action and ability to disintegrate necrotic Pulp, sodium hypochlorite (NaOCl) is the most commonly used solution for root canal irrigation [12, 20]. The concentration and duration of NaOCl determine its characteristics, as well as its toxicity to apical tissues. As a result, for root canal disinfection, lower concentrations of NaOCl should be used in larger volumes, frequencies, and contact durations [12, 20]. The NaOCl concentration and contact time examined in this study were determined using the most frequently used concentration of NaOCl by Ghanaian dentists and the higher concentrations used in other studies [13, 21, 22].

Gazzaneo et al. (2019) analyzed the decontamination ability of different concentrations of NaOCl (6% and 2.5%) on intracanal bacterial during chemo-mechanical preparation using a single-file technique varying the volume, concentration, and retention time of sodium hypochlorite (NaOCl) irrigation in comparison with a multifile system [21]. It was discovered that in the 2.5% concentration, the volume used was decisive for the disinfection of the channels.

There was an overall decrease in the colony-forming units (cfu/ml) among the concentrations of NaOCl used in this study. This aligned with many other findings [14] [23, 24], which confirmed a more than 90% reduction in colony-forming units from root canals; also, a previous study indicated that, in comparison to the 2.5% of NaOCI solution, 5.25% solution of NaOCI cleaned the root canal substantially better [25].

The effectiveness of NaOCl increases with increasing concentration. [26] In this study, there was a 100% reduction of colony-forming units after treatment in the groups irrigated with the higher concentrations (5.2% and 2.6%) of NaOCl.

Numerous research studies have studied the effects of varying NaOCI concentrations on antibacterial activity, tissue breakdown, debris removal, and post-operative pain [15, 16]. Despite these investigations, there is still no agreement on the appropriate NaOCI concentration. Siqueira et al. [17] found that all concentrations of 1%, 2.5%, and 5.25% NaOCl were efficient against bacteria, and the effect increased with larger concentrations. The 5.25% and 2.5% concentrations of NaOCl reduced colony-forming units by 99.9% and 99.8%, respectively, congruent with our findings. The ability to reduce colony-forming units was not significantly different between the three NaOCl solutions tested in Siqueira et al.’s study [12]. Ayhan et al. [18] examined the antibacterial effects of 0.5% and 5.25% NaOCl on various microbes and found that the 0.5% concentration was less effective.

The difference in percentages between this study’s findings and the others above may be due to variations in the procedure used. For example, Siqueira’s looked at the effects on E. faecalis, while this study looked at all isolated microbes. Additionally, Siquera’s study was an in vitro study that looked at colony-forming units of the reduction of the study’s intracanal bacteria by instrumentation with 1%, 2.5%, and 5.25% sodium hypochlorite (NaOCl).

The average cfu/ml before and after treatment showed a significant difference (p < 0.001). Furthermore, the lower concentrations (0.5% and 1.0%) of NaOCl, though effective, were not as effective as the higher concentrations (2.6% and 5.2%) that eliminated all microorganisms post-treatment.

The study identified a wide variety of microbial species before treatment, with obligately anaerobic species being the most prevalent. After treatment, no microorganisms were isolated for the groups with higher concentrations(2,5 and 5.2%). The two lowest concentrations (0.5% and 1.0%) of NaOCl eliminated bacteria in all but two teeth. These two teeth had *Streptococcus mitis* and *oralis* isolated, respectively. Similar to this, Siqueira et al.’s [12] analysis of the chemo-mechanical reduction of the bacterial population in the root canal following instrumentation and irrigation with sodium hypochlorite at concentrations of 1%, 2.5%, and 5.25% concluded that while using NaOCl in low concentrations may significantly reduce endodontic infection, it may not always dissolve all pulpal remnants promptly. The effectiveness of root canal cleaning increases with increased NaOCI concentration, according to a different study that histologically assessed the efficacy of debris removal by activating 2.5% and 5.25% NaOCI utilizing laser, ultrasonic, and intracanal heating techniques.

After chemo-mechanical methods, root canal samples that test positive for bacterial growth often contain an average of 1 to 5 bacterial species [5, 27, 28]. *S*.*oralis* and *S*.*mitis* are among the most common gram-positive bacteria persisting after endodontic treatment and intracanal disinfection procedures [29]. This might be the reason why these bacteria were recovered in 2 teeth in the groups irrigated with 0.5% and 1.0% NaOCl, respectively. The microorganisms isolated from the teeth requiring root canal treatment were highly sensitive to the selected concentrations (0.5%, 1.0%.2.6%,5.2%) of NaOCl used; hence, incorporating NaOCl in root canal treatment will produce better endodontic results.

### Effect of the selected (0.5%, 1.0%, 2.6%, 5.2%) concentrations of NaOCl on post-operative pain

Post-operative pain following endodontic treatment remains common, with a 3–58% prevalence [29, 30]. In this study, 16.7% of the participants experienced mild post-operative pain within 24 hours, which resolved within 48 hrs.

The reduction in the pre-treatment pain levels and the low incidence of post-treatment pain observed in this study show that mechanical debridement and irrigation of the root canal with sodium hypochlorite can remove necrotic tissues, which is a reservoir of dead microbes and their products within the lumen of the root canal. These products reach the periapical region and act as a source of infection, leading to inflammation and pain.

According to Jaclyn Pak et al.’s [31] systematic review of the prevalence of pain before and after root canal therapy, the frequency and severity of pain significantly decreased. The degree of root canal-related pain was moderate before treatment but significantly reduced the following day.

In this study, using Wilcoxon signed-rank test analysis, the groups irrigated with 1.0% (p = 0.005) and 2.6% (p = 0.005) concentrations of NaOCl showed a significant reduction in pain rating after the endodontic treatment. Though there was a reduction in the pain rating pre and post-treatment in the groups irrigated with 0.5% and 5.2% concentrations of NaOCl, it was not significant. This varies from reports by  Farzaneh et al. [32] who reported that 5.25% NaOCl had significantly lowered post-operative pain incidence compared with those who had 2.5% NaOCl during the first 72 hrs following treatment (*p* = 0.021). The variations in the preoperative pulpal and periapical state of the teeth and the techniques used may cause the discrepancy between the results of the previous study and the current one.

However, the results agree with the findings that showed that at 24 hours, the overall incidence of discomfort was 42.2% [33]. Even though the lower concentrations reported a lower incidence of pain (37.8%) than the higher concentration group (46.7%), the difference was not statistically significant (P >.05). At all of the assessed time intervals, there was no discernible difference in the post-operative pain intensities between the two groups [33]. Their study procedures used methods similar to this current study, and root canal treatment was performed during two visits, with calcium hydroxide interappointment antimicrobial dressing used to prevent germs from recovering and growing in the residual root canal space. The study participants experienced post-operative pain within 24–48 hours following their initial treatment session; there was no pain after this period.

Within the limitation of this study, utilization of the 2.6% and 5.2% concentrations of NaOCl as irrigants led to the complete elimination of the colony-forming units and the microbes in the root canals of the teeth. This shows that using concentrations of NaOCl above 1.0% and up to 2.6% will effectively eliminate root canal bacteria and minimize post-operative pain experience If root canal treatment is done using standard mechanical debridement and irrigation procedures.

### Conclusion and recommendation

The findings of this study suggest that root canal therapy using NaOCl effectively reduces microbial load and alleviates pain, eliminating all microorganisms in the 2.6% and 5.2% concentration groups showing no isolates post-treatment after treatment. Within the limitation of this study, root canal treatment done using standard mechanical debridement and irrigation procedures with appropriate concentrations of NaOCl above 1.0% and up to 2.6% have the potential to adequately disinfect the root canals and enhance clinical outcomes. The highlights the importance of selecting the appropriate concentration of NaOCl that has efficacy in microbial reduction or pain management.
